# An interactive visualization tool to explore the biophysical properties of amino acids and their contribution to substitution matrices

**DOI:** 10.1186/1471-2105-7-329

**Published:** 2006-07-03

**Authors:** Blazej Bulka, Marie desJardins, Stephen J Freeland

**Affiliations:** 1 Department of Computer Science and Electrical Engineering, University of Maryland, Baltimore County, 1000 Hilltop Circle, Baltimore, MD 21250, USA; 2 Department of Biological Sciences, University of Maryland, Baltimore County, 1000 Hilltop Circle, Baltimore, MD 21250, USA

## Abstract

**Background:**

Quantitative descriptions of amino acid similarity, expressed as probabilistic models of evolutionary interchangeability, are central to many mainstream bioinformatic procedures such as sequence alignment, homology searching, and protein structural prediction. Here we present a web-based, user-friendly analysis tool that allows any researcher to quickly and easily visualize relationships between these bioinformatic metrics and to explore their relationships to underlying indices of amino acid molecular descriptors.

**Results:**

We demonstrate the three fundamental types of question that our software can address by taking as a specific example the connections between 49 measures of amino acid biophysical properties (e.g., size, charge and hydrophobicity), a generalized model of amino acid substitution (as represented by the PAM74-100 matrix), and the mutational distance that separates amino acids within the standard genetic code (i.e., the number of point mutations required for interconversion during protein evolution). We show that our software allows a user to recapture the insights from several key publications on these topics in just a few minutes.

**Conclusion:**

Our software facilitates rapid, interactive exploration of three interconnected topics: (i) the multidimensional molecular descriptors of the twenty proteinaceous amino acids, (ii) the correlation of these biophysical measurements with observed patterns of amino acid substitution, and (iii) the causal basis for differences between any two observed patterns of amino acid substitution. This software acts as an intuitive bioinformatic exploration tool that can guide more comprehensive statistical analyses relating to a diverse array of specific research questions.

## Background

Molecular biology has made great progress in observing and quantifying the patterns by which amino acids exchange for one another within protein sequences over time. A key motivation here has been to create amino acid substitution matrices (such as the PAM and BLOSUM matrix families), which lie at the heart of mainstream bioinformatics procedures, from algorithms that determine whether [1] and how exactly [2] two proteins are homologous, to those that predict protein tertiary structure by comparison with known folds [3]. However, these matrices represent generalized patterns of change "averaged" across all proteins: although they typically encompass the idea that patterns of substitution will vary with evolutionary distance, other systematic sources of variation are overlooked. An increasing literature supports the idea that this generalization may compromise the sensitivity of sequence comparison for various specialized subsets of proteins (e.g., for particular protein families [4-8], or for genomes that have evolved under unusual mutation biases or selection regimes [9-11]). Thus a worthy challenge is to seek the underlying ontology that can link individually derived, specialized models of amino acid substitution into a common framework: if we can ultimately replace generalized patterns of observed change with a flexible, quantitative model of amino acid substitution, then this offers significant potential to increase the sophistication of standard bioinformatics procedures. Such research may in fact be viewed as a subset of current efforts to find a general, chemical ontology for bioactivity (e.g., [12-14]) where researchers face the same challenge of unifying diverse observations into a model that predicts molecular interactions from first principles.

In this context, it has long been understood that amino acid substitution matrices reflect a combination chemical and evolutionary factors: most intuitively the biophysical properties (known within chemical disciplines as "molecular descriptors") of the amino acids [15,16] and the mutational distance of their encodings within the genetic code [5,17,18]. However, establishing accurate, quantitative connections between the outcomes of molecular evolution and amino acids' molecular descriptors remains a complex issue under active research (e.g., [19-21]).

In this context, Nakai *et al*. created an innovative database, the AAindex [22], comprising both amino acid substitution matrices (20 × 20 matrices in which each element reflects some measure of the exchangeability of a pair of amino acids) and amino acid indices (vectors of 20 elements, each element being a value that describes some physiochemical property such as size or hydrophobicity, for one of the twenty amino acids encoded by the standard genetic code). In a later publication that expanded this database, Tomii and Kanehisa [23] suggested procedures for correlating any amino acid molecular descriptor with an observed exchange rate (e.g., substitution matrix) and for clustering indices together by similarity.

This latter technique of index clustering, is especially useful when exploring the relationship between indices, given that properties of widespread interest have often been measured in many different ways by different researchers. (For example, the latest version of the AAindex database [24] contains 29 different measurements of a property that contains the term "hydrophobicity" in its description.) Moreover, this comparison allows easy visualization of non-intuitive correlations (e.g., hydrophobicity and volume). The authors applied similarity-based methods to their AAindex database to build a *minimum spanning tree*: a graph-theoretic structure that connects discrete elements together based on similarity, by minimizing the overall sum of the distances of the direct connections. The result is a data structure in which elements are grouped together based on similarity (a detailed description and justification is given in the work of Tomii and Kanehisa who first applied this methodology to visualizing amino acid similarity [23]). This minimum spanning tree showed the underlying structure (clustering) for the 402 indices of their database. Since this time, numerous further indices and matrices have been developed: some have been incorporated into updates of the AAindex, while others remain isolated in the scientific literature (e.g., [10,25]).

In this context, we have developed free, user-friendly, publicly available web-based software that enables researchers to repeat and extend the ideas of Nakai *et al*., [22] and Tomii and Kanehisa [23] using interactive data visualization. We thus present the Amino Acid Explorer, a web tool that facilitates quantitative exploration of similarity between physiochemical properties of amino acids and their evolutionary dynamics. Our tool allows users to explore the similarity between any of the 83 matrices and any subset of the 494 indices housed by AAindex version 6.0, and to include any custom index or matrix (e.g., from recent scientific literature or from unpublished research, as a matrix derived from an alignment of proteins in a particular functional class, or an index derived by combining several physiochemical properties). We have embedded this analysis tool within a comprehensive web context: both a moderated user forum  in which to discuss problems, findings or questions and an open wiki  in which the community of those researching the interface of biochemistry and protein evolution may contribute their knowledge.

## Implementation

**Figure 1:**
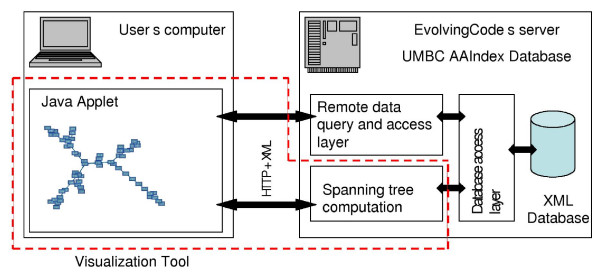
Overview of Amino Acid Explorer Architecture.

Our web tool, which may be accessed at , comprises two major parts: one client side, one server side. The client side consists of the graphical interface that runs as a Java applet within a user's browser. The server side (residing on ), is a web application that performs all computations on the data, and is part of a larger computational infrastructure created around UMBC AAIndex database. Figure 1 shows an overview of our tool's architecture. Additionally, a short paragraph describing UMBC AAIndex database is located at the end of this section.

### User interface and visualization

The user interface of our tool is a Java applet that runs in a user's browser. It allows the user to (i) select any subset of the AAIndex indices (or custom indices) to be clustered using the minimum spanning tree method, (ii) choose an appropriate distance calculation method (to be used during the spanning tree computation), and (iii) choose a matrix or matrices to compare with the indices of a spanning tree.

Specifically, having built a spanning tree, the application can compute distances between all the indices in this tree and a user defined matrix; it displays these distances by shading the elements of the spanning tree with a color-coded scale. Additionally, it can use a second color-coded scale to display which of two user-defined matrices each index of the spanning tree is closest to (in other words, what makes these two matrices different from one another in terms of the indices under consideration?).

#### Drawing the spanning tree

Graph drawing and visualization are currently open research topics in computer science [26]. Although an agreed method exists for creating the graph (calculating a spanning tree), finding an optimal spatial positioning for nodes and drawing edges in a readable way (e.g., grouping nodes that are directly connected together, while minimizing crossed edges) remain active areas of research. A large number of different software packages implement a variety of state-of-the-art graph drawing methods, which differ significantly in speed, quality of the drawing, and interactivity (i.e., allowing the user to influence the final shape of the graph being drawn). Our visualization tool uses a slightly modified form of the open source-package TouchGraph [27] to render the minimum spanning tree that was computed server-side. (Modifications to the original TouchGraph code are limited to changes that redefine the default parameters for flexibility of the edges, and minor modifications required to integrate the code into our applet.) A full description of TouchGraph can be found at their web site; in essence, it uses an iterative "force-based layout" algorithm (in which nodes each projects a force that repel other nodes, while edges act like springs that can be compressed or stretched) to move, though a series of incremental improvements, from a random graph layout to an optimal representation. The whole incremental process is visible, and the user can intervene at any point by dragging nodes to locations that seem to be better suited. In our application, this is most likely to be useful when users request a spanning tree for a large set of amino acid indices, under which conditions the force-based layout may become stuck at a local optimum, visible to the user as a representation in which one or a few key edges cross one another.

#### Visualizing distances between a matrix and a set of indices

Our application represents the distances between matrices and indices in two modes. In the first mode, each node in the spanning tree (representing a single amino acid index) is color-coded to represent its measured similarity to a single, user-defined reference matrix. The color scale runs from blue (most distant) to red (most similar). Distances are measured as described below. The second mode (*differential mode*) shows how two substitution matrices differ in terms of the amino acid indices of a spanning tree. This mode uses a color-coded scale to denote which of two matrices is closest to each node (index). In the figures shown here, the color scale is green (matrix 1) to brown (matrix 2) so as to avoid any confusion with Mode #1 described above. The degree of color saturation denotes the magnitude of the difference (i.e., strong colors indicate that the two matrices are very different in terms of this index).

### Computations

All significant computation for this tool occurs on the server-side, because it often involves most or all of the data stored in the database (thus transfer to a client-side applet could take prohibitive time for users with low-bandwidth connections).

#### Computation of a minimum spanning tree

The software calculates a minimum spanning tree using Prim's algorithm, as described by Cormen *et al*. [28]. Since this algorithm minimizes the total sum of distances between directly connected indices, the definition of distance here is of prime importance. Tomii and Kanehisa [23] used a statistical correlation measure between two indices (each is a vector of 20 numbers representing an amino acid property). Our software allows users to employ this metric, but also to explore another notion of distance, namely Euclidean distance (calculating distance between two indices as distance between two points in 20-dimensional space). This approach is often taken to compare normalized vectors in multi dimensional spaces [29]. More generally, our software allows users to restrict the set of amino acids that are taken into account when calculating distance (e.g., it is possible to consider only hydrophobic amino acids, or only those encoded by GC-rich codons), whichever metric of distance is being used.

#### Computation of distance between a matrix and a set of indices

In order to compute the distance between a matrix and a set of indices, our software uses the correlation method described by Tomii and Kanehisa [23]. This method first converts each index (a vector of 20 values, one for each amino acids) into a matrix by calculating the simple arithmetic distance between each pair of amino acids, as defined by the index. It then calculates the correlation coefficient between these two matrices. While the Euclidean distance method may be used to build a minimum spanning tree of indices, which have been normalized to facilitate direct comparison, this method would is inappropriate for matrix/index comparisons because matrix values have not been normalized (i.e., matrix elements may extend beyond the interval from 0 to 1 and thus Euclidean distance between any one element of an index and elements of a matrix would be misleading. Linear normalization of matrix elements would itself be inappropriate since many matrices, such as the PAM series, comprise values that are expressed in logarithmic units). Therefore, our software always uses the Tomii and Kanehisa method of simple correlation to compare a matrix with an index. If the user has selected only a subset of the 20 amino acids for tree building, then calculations of distance between a matrix and the indices of a spanning tree consider only the appropriate subset of matrix elements.

### UMBC AAIndex database

We created the UMBC version of the AAIndex database as a local version of the original AAindex data (created by GenomeNet Japan [30]) to facilitate the manipulations required by our interactive software. Specifically, our local implementation converted all data of the original AAindex to XML format, generated interfaces that enable precise local and remote access to all aspects of the database, and normalized all amino acid index data.

XML is a standardized language that is designed to simplify sharing of information among independently created systems. In particular, it is easily readable by machines (there are many code libraries that allow access to XML data by programs written in almost any programming language), and thus facilitates conversions to other languages, both to formats that are intended to be read by humans (e.g., web pages or PDF files) and to other computer formats. Our UMBC AAIndex database allows direct user access via internet either in "raw" form (plain XML data) or transformed to a web page that is designed to be easily read by a human. In the former capacity, our implementation of this database has been designed for simple access by either programs residing on our server, or by simple HTTP requests from remote machines. When bandwidth for data transfer is an issue for some third-party users, our architecture also allows deployment of programs directly at the server for a more direct access. Both of these latter points reflect our aim to facilitate other researchers who would like to expand and improve the functionality we offer for the AAindex data.

The indices in the database have been normalized by linearly scaling all the values of each index from 0 (the smallest value of the original index) to 1 (the greatest value of the original index). This simplifies and makes more intuitive the comparison of values between different indices, which may originally have had values expressed using different units. (Note that this normalization does not influence the results obtained by the correlation coefficient method used by Tomii and Kanehisa [23], which may be reproduced exactly by our software in a matter of seconds.)

## Results

Here we present three simple, example analyses to illustrate the types of exploration that our software allows. Each illustrates a conceptually different question that the tool reduces to a simple "point and click" exercise. We have chosen to focus on the relationship between biophysical properties of amino acids, patterns of molecular evolution, and the structure of the standard genetic code. However, it would be trivial to find an equivalent set of example analyses that focused on protein folding or homology searching. Indeed, our visualization software can be used to investigate any area of bioinformatics that builds on understanding how amino acids' molecular descriptors influence the patterns by which amino acids substitute for one another during evolution.

**Figure 2:**
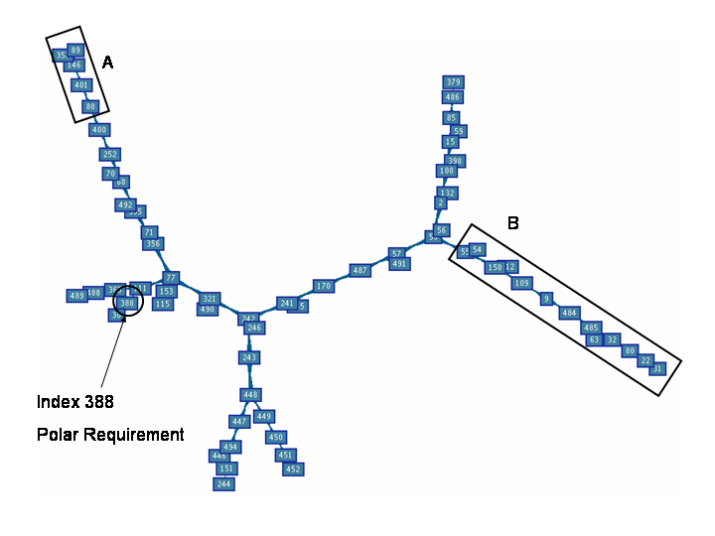
**A minimum spanning tree of size, charge and hydrophobicity for the 20 amino acids of the standard genetic code**. Specifically, this tree is built from the 67 amino acid indices that contain the words "hydrop" and/or "polar," "size," "volume," "charge," and "electr" as part of their description. This includes most of the indices that relate to the general concepts of amino acid size, charge, and hydrophobicity. Boxes A and B represent "natural" clusters formed by the minimum spanning tree of charge and size, respectively.

In Figure 2, we show an analysis (taking approximately 40 seconds to produce) in which we build a minimum spanning tree of indices relating to amino acid size, charge, and hydrophobicity. Interestingly, while measures of charge and size form coherent units (boxes A and B respectively), the more numerous measures of hydrophobicity form three major branches. Notably, index 388, Polar Requirement [31], is a measure of amino acid polarity that has been used extensively in developing evidence for the idea that the pattern by which amino acids were assigned to codons within the standard genetic code results from natural selection to minimize the change in amino acid hydrophobicity caused by point mutations [32-35]. Although this minimum spanning tree emphasizes the legitimacy of treating Polar Requirement as a measure of hydrophobicity (its authors originally introduced the metric as an estimate of stereic affinities between nucleotides and amino acids [36]), the tri-partite spanning tree for the concept of hydrophobicity illustrates the potential dangers of over-emphasizing any one measure of hydrophobicity. In this context, it is helpful to note that a second "branch" of amino acid hydrophobicity measures includes Kyte and Doolittle's [37] "hydropathy" (index 151) which is also strongly reflected by the codon assignments of the standard genetic code [32].

**Figure 3:**
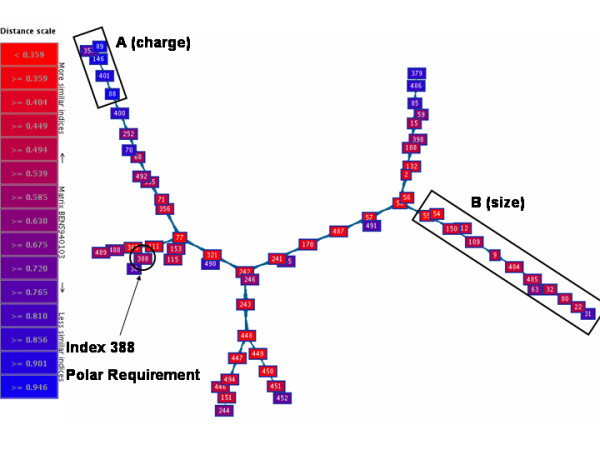
**The minimum spanning tree recolored to reflect distance to a PAM matrix**. Specifically, the minimum spanning tree of size, charge, and hydrophobicity (Figure 2) is recolored to indicate the similarity of each amino acid index to the PAM74-100 amino acid substitution matrix [5].

In Figure 3, we show a second analysis (taking approximately 5 seconds to produce, given the tree of Figure 2) in which we measure the similarity of each index in our original minimum spanning tree to a classic amino acid substitution matrix: the PAM 74–100 [5]. Here we see that generally, measures of amino acid hydrophobicity correlate well with observed patterns of amino acid substitution, though interestingly, Polar Requirement is by no means the strongest of these (an observation pertinent to the debate over cause and effect of hydrophobicity as a dominant explanatory variable of generalized amino acid substitution patterns [38,10]). Amino acid volume shows some correlation with substitution patterns, but charge (as measured by these indices) is by far the least related property. This provides a quick, empirical justification for the general patterns predicted, for example, by Grantham [4]. It also matches analyses of which fundamental amino acid properties are reflected within the codon assignments of the standard genetic code [32,37].

**Figure 4:**
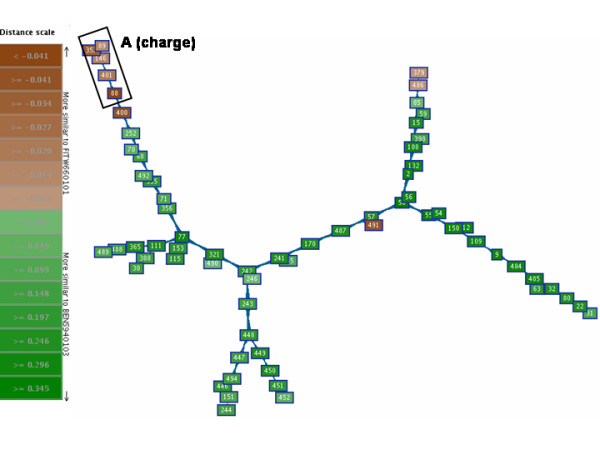
**The minimum spanning tree recolored to show each index's similarity to one of two substitution matrices**. Specifically, the spanning tree of size, charge, and hydrophobicity (Figure 2) is recolored to indicate whether each amino acid index is more highly correlated with the PAM74-100 amino acid substitution matrix (green) or a matrix of amino acids' proximity within the standard genetic code [8] (brown).

In Figure 4, we show a further analysis (taking approximately 10 seconds in total to produce, given the tree of Figure 1) that explores how the PAM74-100 matrix differs from Fitch's matrix of "mutational distance between amino acids within the standard genetic code" [6] in terms of amino acid size, charge and hydrophobicity. We find that in general, measures of hydrophobicity and volume are closer to the PAM matrix (i.e., are more correlated with observed patterns of amino acid substitution), whereas the small cluster of amino acid indices relating to charge correlate more strongly with the genetic code based matrix. On a simple level, this quick analysis shows that the standard genetic code does indeed contain an element of non-random codon assignments with respect to amino acid charge, as reported in an erratum by Haig and Hurst [40] that replaced their initial rejection of such a link [32]. At a deeper level, these results are germane to debates over the flow of causality that links amino acid physiochemical properties to observed patterns of amino acid substitution within proteins – the mainstream view is that physiochemical properties dominate the pattern by which amino acids substitute for one another, particularly over large stretches of evolutionary time [5]. However, there has been some debate as to whether (and to what extent) such patterns can be caused by neutral evolution that substituted amino acids based on their mutational proximity within the standard genetic code, given that the code is non-randomly organized with respect to key amino acid properties [10,41,38]. Our quick analysis indicates that physiochemical considerations really are, in fact, more important to long-term protein evolution than can be explained by codon assignments (in that the physiochemical properties are more strongly correlated with observed substitution patterns than with mutational distance within the genetic code; i.e., physiochemical similarity comes to dominate patterns of substitution as evolution proceeds).

This same feature of the AAIndex Explorer tool could equally well be used to quickly visualize which properties (and which amino acids) are responsible for the difference between any two substitution matrices (e.g., between a "generalized" or global model of amino acid substitution, as found in a PAM or BLOSUM matrix, and any observed pattern of interchange within a specific protein family or phyletic lineage).

## Conclusion

In this paper, we present software that facilitates rapid, interactive exploration of data pertaining to three interconnected topics: (i) the multidimensional molecular descriptors of biochemical properties for the twenty proteinaceous amino acids, (ii) the correlation of these biophysical measurements with observed patterns of amino acid substitution (i.e. substitution matrices), and (iii) the causal, biocehmical basis for differences between any two observed patterns of amino acid substitution. This software acts as an intuitive bioinformatic exploration tool that can guide more comprehensive statistical analyses relating to a diverse array of specific research questions.

## Availability and requirements

Project name: Amino Acid Explorer

Project home page: 

Operating system(s): Platform independent

Programming language: Java

Other requirements:

• Use via EvolvingCode's website

○ Web browser (tested with Internet Explorer, Netscape and Mozilla under Windows and Linux, Safari under Mac OS X 10.3.9)

○ Java 1.4.2 plug-in for the web browser (or higher version)

• Full installation on an independent server

○ Java 1.4.2 plug-in for the web browser (or higher version) on the client side

○ JDK 1.4.2 environment on the server

○ XML Database compliant with XML:DB API (tested with eXist database)

○ Servlet Web Container matching Servlet API 2.4 specifications (tested with Tomcat 5.0.28)

○ Xalan XSLT processor

License: Apache-style open source license

Any restrictions to use by non-academics: None

## Authors' contributions

**BB **created the local implementation of the AAindex database, including XML schemas, coded the spanning tree software, and wrote the computer science aspects of this paper. **SJF **came up with the concept of this software, supervised software development, and wrote the biological portions of this paper. **MdJ **supervised and provided technical expertise for the computer science involved in this project
